# Determination of Cotinine, 3′-Hydroxycotinine and Nicotine 1′-Oxide in Urine of Passive and Active Young Smokers by LC-Orbitrap-MS/MS Technique

**DOI:** 10.3390/molecules29153643

**Published:** 2024-08-01

**Authors:** Magdalena Szumska, Paweł Mroczek, Krystyna Tyrpień-Golder, Beata Pastuszka, Beata Janoszka

**Affiliations:** 1 Department of Chemistry, Faculty of Medical Sciences, Medical University of Silesia, 40-752 Katowice, Poland; d201099@365.sum.edu.pl (P.M.); ktyrpien@sum.edu.pl (K.T.-G.); 2 Research and Implementation Center Silesia LabMed, Medical University of Silesia, 40-752 Katowice, Poland; betty.p2308@gmail.com

**Keywords:** nicotine metabolites, cotinine, passive-smoking, urine, liquid chromatography, mass spectrometry

## Abstract

Tobacco smoke is probably the most significant factor conducing to toxic xenobiotics exposure to humans. The aim of the study was to develop a rapid and sensitive method for the determination of selected nicotine metabolites in urine of tobacco smokers and passive smokers. The method for removing protein and extracting the metabolites involved the centrifugation of urine with acetonitrile. Cotinine, trans-3′-hydroxycotinine, and (2′S)-nicotine 1’-oxide in the supernatant were determined using the LC-Orbitrap-MS/MS technique, with the selected ion monitoring (SIM) and parallel reaction monitoring (PRM) modes used. The recovery of these analytes added to the urine samples ranged from 72% to 101%. Repeatability and reproducibility were less than 3.1% and 10.1%, respectively. The study was carried out among medical students. The group was selected as representatives of young people and who as future physicians should be more aware of the effects of nicotine use. Concentration levels of cotinine and *trans*-3′-hydroxycotinine determined in ng/mL in the urine of cigarette smokers were 70- and 58-fold higher, respectively, compared to passive smokers. Higher concentrations were recorded in the urine of those passively exposed to tobacco smoke than in non-smokers, confirming that passive exposure to tobacco smoke is not harmless to the human body. However, no significant differences were observed in the concentration of (1′S,2′S)-nicotine 1′-oxide in the samples of individuals from various groups.

## 1. Introduction

Tobacco consumption is one of the most serious public health problems, causing a multitude of chronic diseases such as cancer, cardiovascular disease, and chronic obstructive pulmonary disease. Smoking one cigarette reduces life expectancy by approximately 11 minutes. Cigarette smoke contains over 5000 different chemicals, among which 63 are carcinogenic, as reported by the International Agency for Research on Cancer (IARC); moreover, 11 of these are known human carcinogens, increasing the risk of numerous diseases affecting many organs [1,2,3]. 

Nicotine is one of the main addictive constituents of tobacco and the main component of cigarette smoke. However, it is not a carcinogen, but it is still a highly toxic compound with an LD_50_ within the range of 6.5 to 13 mg/kg of body weight. Since nicotine has a relatively short half-life (1–2 h), it is not a useful biomarker for assessing tobacco smoke exposure, especially when estimating the impact of second-hand smoke [4].

Biomarkers of tobacco smoke exposure are considered indicators of the absorbed dose. They can provide objective measures to assess the impact of tobacco products’ impact on the general population and their health consequences. In this group of biomarkers, components of tobacco smoke or their biotransformation products and adducts with DNA and proteins, including thiocyanates, carbon monoxide or its adduct with hemoglobin, nicotine and its metabolites, and hydroxy-PAHs (including 1-hydroxypyrene or acrylamide metabolites) can be found [5]. 

Nicotine metabolites with longer half-lives are most often used as biomarkers of exposure to tobacco products and can be detected in different biological fluids such as plasma, serum, urine, and saliva [1,4,6,7]. The total amount of absorbed nicotine metabolized to cotinine ranges from 70–80%, and approximately 10% of this alkaloid is excreted in urine. Nicotine is mainly metabolized by the P450 enzyme system, which leads to the formation of six primary metabolites: cotinine, *trans*-3′-hydroxycotinine, norcotinine, nornicotine, cotinine oxide, and nicotine oxides [3,8]. 

Cotinine is the most widely analyzed biomarker of exposure to tobacco smoke and has been successfully used for the study of smoking habits and passive exposure, making it possible to distinguish smokers from non-smokers and passive smokers. Cotinine has a fairly long elimination time from the body and can be detected within days after exposure to tobacco smoke. According to the literature, the biological half-life of cotinine ranges from 8 to even 30 h [2,8,9]. Concentrations of cotinine in urine, saliva, and blood samples are strongly associated with nicotine exposure [10]. 

Cotinine is the primary metabolite of nicotine. However, it has been estimated that only approximately 10% to 15% of cotinine is found in smokers’ urine, as most cotinine is converted into other metabolites in the process of hydroxylation to *trans*-3′-hydroxycotinine. *Trans*-3′-hydroxycotinine has an average half-life of 4 to over 8 h and is another suitable biomarker of exposure to nicotine. Its half-life is similar in both blood and urine samples. *Trans*-3′-hydroxycotinine accounts for approximately up to 40% of the absorbed nicotine dose [2,3]. Measuring the ratio of cotinine to *trans*-3′-hydroxycotinine makes it possible to investigate the metabolism of nicotine. Research has confirmed the usefulness of determining the molar ratio of these two metabolites as a marker of CYP2A6 isoenzyme activity, which determines the rate of metabolization of nicotine [11,12]. Another primary nicotine metabolite is nicotine -N-oxide. It is estimated that approximately up to 7% of nicotine is metabolized to nicotine-N-oxide. This metabolite can be reduced back to nicotine in the digestive tract [2]. Biomarkers of tobacco smoke exposure are considered indicators of the absorbed dose and, according to the current state of knowledge, they can be components of tobacco smoke or their biotransformation products and adducts with DNA and proteins [2,4]. They can be detected and analyzed in different biological matrices such as blood, urine, saliva, and hair [1,7,13,14]. Due to the invasive nature of blood sampling, other non-invasively collected biological samples are often used with great success. For example, urine samples are much easier to collect and contain higher concentrations of nicotine metabolites compared to other biological fluids. This property also makes urine samples the most sensitive matrix for evaluating second-hand tobacco exposure [1,3,13].

Over the years, many analytical methods have been developed for the determination of nicotine metabolites. The most frequently applied methods for the determination of tobacco smoke exposure biomarkers are gas chromatography (GC) and liquid chromatography (LC) coupled with mass spectrometry (MS), but immunological methods can also be found [2,4,6,15]. Immunochemical tests can be used for screening. Despite their high sensitivity, the specificity of these methods is low. For example, cotinine quantification results may be overestimated due to cross-reactions with other nicotine metabolites [16,17].

Chromatographic techniques, especially in combination with mass spectrometry, allow the separation and determination of various metabolites of nicotine due to their high specificity. Before introducing mass spectrometry to chromatographic analysis, different detectors were used for biomonitoring. GC with flame ionization or the more sensitive and specific nitrogen–phosphorus detector (NPD) was applied [16,18,19].

High performance liquid chromatography (HPLC) with an ultraviolet absorption detector has been used to detect nicotine and its metabolites in biological fluids but is now increasingly being replaced by LC methods connected with mass spectrometry [6,16,20].

Biological samples require some pretreatment steps, which include solid-phase extraction (SPE), acidic precipitation, or simple liquid–liquid extraction. The cleanup procedures vary depending on the chosen biological fluid, as these samples have different matrix content that can influence the determination process. However, when it comes to noninvasive samples like urine, the most common method to remove proteins/salts is organic solvent precipitation using acetonitrile or methanol [3,15,21]. Some authors use acetone precipitation pretreatment for urine samples pointing to lower toxicity and faster evaporation of that solvent compared to acetonitrile, water, or methanol. Recovery rates for sample pretreatment procedures vary depending on the procedure used. For solid phase extraction (SPE), they are in the range of 57 to 124%; for liquid–liquid extraction, they range from 53 to 117%, and organic solvent precipitation leads to recoveries in the range of 89 to 105% [3,15,21].

Efficiently examining a large number of samples to study smoke exposure in the general population requires a simple and fast sample preparation and analysis procedure. Although the GC-MS technique is described as a sensitive one, the running time of analysis is much longer (15–30 min) compared to LC-MS (6–14 min). LC-MS/MS is the most promising technique for nicotine metabolite analysis as it allows the determination of a wide range of analytes regardless of their properties. It enables adequate analytical separation and better-quality mass spectral data, leading to enhanced detection limits. For nicotine metabolite analysis, a triple quadrupole MS system is mostly chosen, and the determination of target compounds is performed under multiple reaction monitoring mode with positive electrospray ionization using a C18 or HILIC analytical column [22,23,24]. In analytical procedures for elution, an isocratic or gradient flow of water and acetonitrile with formic acid (for C18 column methods) or ammonium acetate (for HILIC column methods) is most commonly used. The column temperature between 30 and 40 °C is applied in most LC-MS methods to avoid peak shifting [8,25]. Although the methods used for the determination of nicotine metabolites in biological samples such as urine are sensitive, their quantification can present analytical difficulties. One of the analytical obstacles is the matrix effect. To minimize the matrix effect of biological samples on the results of the analysis, the use of isotopically labeled internal standards is recommended [4,22]. 

Using LC-MS/MS techniques in monitoring tobacco smoke health hazards makes it possible to detect even very low levels of exposure, giving scientists a tool to verify environmental exposure to tobacco connected to passive smoking. Since urine accumulates higher concentrations of nicotine metabolites, it may be the most sensitive matrix in the assessment of passive smoking [3,16]. Passive smoking is estimated to be the cause of approximately 1.0% of worldwide mortality, and this number is increasing every year. It is of extreme importance to implement the sensitive “golden standard” analytical technique to monitor second-hand smoke exposure [3].

Surveys conducted among medical and emergency medicine students have shown that many young adults in this particular group smoke cigarettes and use e-cigarettes as substitutes for traditional cigarettes. Particularly disturbing is the fact that students do not avoid exposure to tobacco smoke, and willingly as well as frequently stay in rooms where cigarettes are smoked by others. This phenomenon is likely due to the demanding nature of medical studies and the need to solve scientific problems in teams, which is a feature of the profession they are being prepared for. Furthermore, another concern is the results of the questionnaire which show that many medical students have limited knowledge of the harmful compounds formed during smoking. Moreover, staying in one room with tobacco smokers is not considered a significant source of exposure to such compounds. These observations were the reason for undertaking research among medical students to prove to them that nicotine metabolites are present not only in the bodies of tobacco smokers, but also in persons passively exposed to tobacco smoke, leading to further health consequences [26]. 

The aim of the study was to develop a rapid and sensitive method for the determination of selected nicotine metabolites in urine, which could be applied to assess the level of exposure to harmful compounds in both tobacco smokers and passive smokers.

## 2. Results and Discussion

### 2.1. Determination of Nicotine Metabolites by LC-Orbitrap-MS/MS

The determination of the three main nicotine metabolites-Cot, 3′-OH-Cot, and 2′S-Nic-Ox-in urine were chosen to evaluate the study participants’ exposure to tobacco smoke. The formulas of these compounds are shown in Table 1.

Using an LC-Orbitrap-MS/MS system equipped with a C-18 analytical column and isocratic elution with an acetonitrile-water solution containing 0.1% HCOOH allowed the components of the standard mixture to be separated and detected in less than 3 min.

All of these molecules are nitrogen-containing heterocyclic compounds. In the presence of acid, they undergo protonation and exist as cationic forms, so determinations by LC technique coupled with mass spectrometry can be carried out in positive ionization mode [27].

Initially, the results of chromatographic separation of nicotine metabolites were obtained in selected ion monitoring (SIM) mode, taking into account the molecular weights of analytes increased by 1 (due to the formation of protonated ions). Although the separation results for the standard mixture allowed for the identification and quantification of the analytes, the results for urine sample extracts indicated potential problems with the identification of nicotine metabolites due to the presence of many other components with similar retention times in biological samples. Supplement 1 shows the chromatogram of the standard mixture (Figure S1A), and chromatograms of urine extracts (Figure S1B–D) recorded using the LC-Orbitrap-MS/MS system in SIM mode. Using this method of determining nicotine metabolites could lead to false positive results.

Therefore, another method was developed for the determination of Cot, 3′-OH-Cot, and 2′S-Nic-Ox using parallel reaction monitoring (PRM) mode. In this method, the highly accurate (with a resolution accuracy of 0.000001 *m*/*z*) masses of molecular precursor ions and the formed product ions were taken into account. The collision energies for obtaining the appropriate product ions from the protonated precursor molecular ions [M+H]^+^ for the nicotine metabolites to be determined are shown in Table 2.

**Figure 1:**
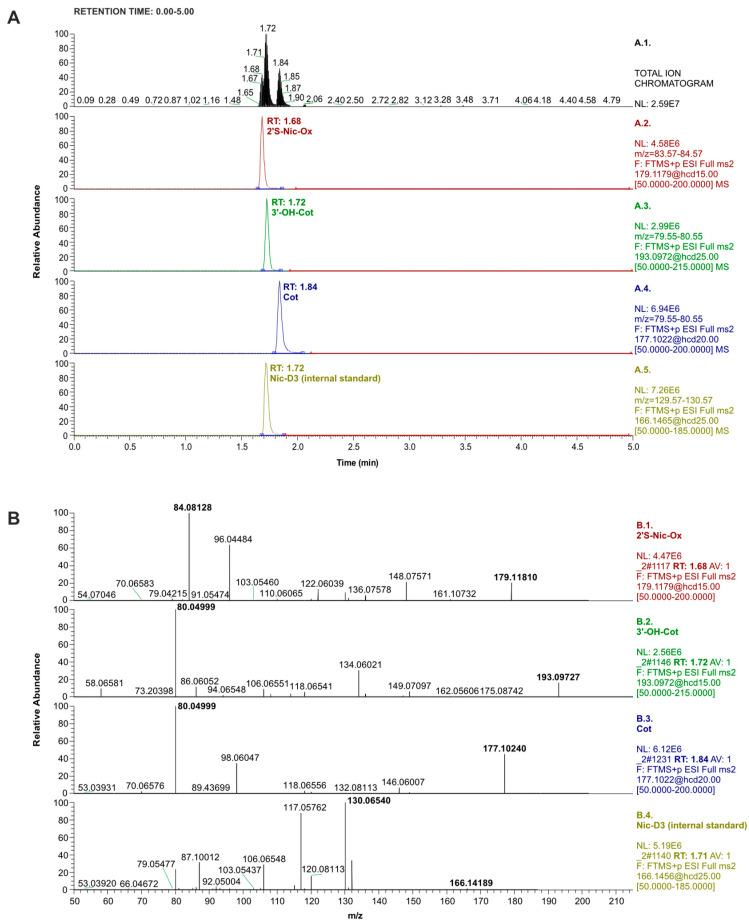
(**A**): LC-Orbitrap-MS/MS chromatograms recorded in total ion mode (top window) and PRM mode (other windows) for nicotine metabolite standards and internal standards (concentration of 0.25 µg/mL; injection: 1 µL). (**B**): Mass spectra recorded in PRM mode of the standards used in the study, shown from the top in order as in Figure 1A, i.e. 2′S-Nic-Ox, 3′-OH-Cot, Cot, and Nic-D3 (internal standard). The appropriate collision energies are presented in Table 2.

The chromatograms obtained as a result of the separation of standard mixture components using the LC-Orbitrap-MS/MS system in PRM mode are shown in Figure 1A. Mass spectra recorded during such analysis are presented in Figure 1B. The determination of Cot, 3′-OH-Cot, and 2′S-Nic-Ox was carried out with the addition of DL-nicotine-(methyl-D3) as an internal standard (IS). The retention time of this compound was the same as Cot, but due to its different molecular precursor mass and product ion mass, Nic-D3 could be used as an IS for the determination of the selected three nicotine metabolites in the standard mixture and urine sample extracts.

Methods for the determination of nicotine metabolites using LC-MS/MS with a tandem or triple quadrupole mass spectrometer often involve monitoring the most intense product ions formed from a protonated molecular ion [4,8,13,21,27]. In such LC-MS/MS systems, this mode of determination is called selected reaction monitoring (SRM) [4,28] or multiple reaction monitoring (MRM) [13,21,25,27,29]. These methods have been used to determine nicotine metabolites, including Cot, 3′-OH-Cot in urine [8,13,25,28,29] and other biological samples, such as plasma [6,27] and saliva [30].

The collision energies of the transitions from precursor ions to product ions in MRM or SRM methods are usually similar to the optimal ones chosen for LC-Orbitrap-MS/MS in our work, although they differ by a few volts in some publications [4,8,13,25,28,29]. The advantage of LC-Orbitrap-MS/MS, however, is that the system allows monitoring of ion masses with high accuracy to 0.000001 *m*/*z*.

### 2.2. Determination of Nicotine Metabolites in Urine Samples

The procedure used to prepare urine samples for the determination of nicotine metabolites included vortexing 0.5 mL of urine with 1 mL of acetonitrile followed by centrifugation of the solution. This stage resulted in protein precipitation. This fast and simple method of sample preparation is commonly used in procedures for determining components of biological samples using sensitive and selective methods such as LC coupled with mass spectrometry [6,7,27,31]. Methanol [27], acetone [4], or aqueous solutions of these solvents are sometimes used instead of acetonitrile [6,7,15,27].

**Figure 2:**
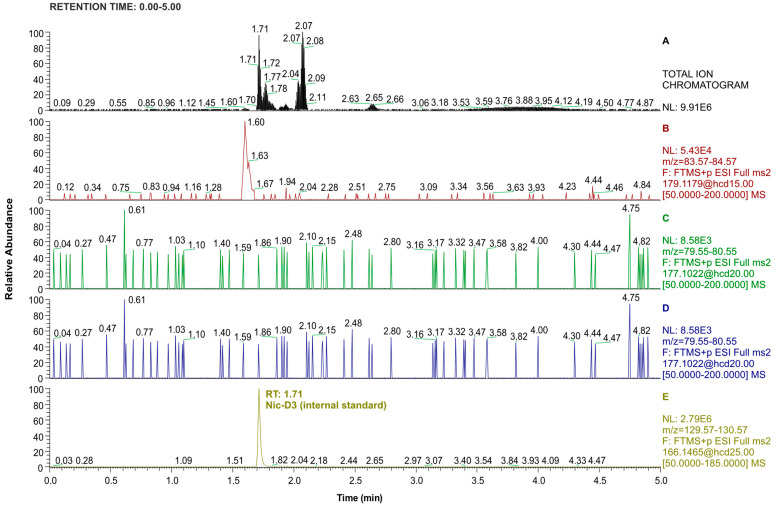
LC-Orbitrap-MS/MS chromatograms recorded in total ion mode (top window) and PRM mode (consecutive windows from the top corresponding to 2′S-Nic-Ox, 3′-OH-Cot, Cot, and Nic-D3) for the urine extract of a non-smoker. Nic-D3 concentration was 0.3 µg/mL (injection volume: 1 µL).

To determine the recovery of the extraction process of nicotine metabolites from urine, a sample of non-smoker urine spiked with standards at three concentration levels (Table 3) was analyzed. This urine sample originally did not contain the analytes selected for testing (Cot, 3′-OH-Cot, and 2′S-Nic-Ox). Chromatograms recorded during the analysis of the non-smoker urine sample without the addition of standards, but with the addition of IS (before the extraction procedure), are shown in Figure 2.

Recovery rates determined from this experiment, shown in Table 3, ranged from 72.2% to 101.4%. These recovery rates are similar to those reported in other studies on the determination of Cot and 2′S-Nic-Ox in urine samples using LC-MS/MS [7,15,22].

The mean concentrations of nicotine metabolites, Cot, 3′-OH-Cot, and 2′S-Nic-Ox, determined in urine samples in our study ranged from: not detected (Cot, non-smokers) to 3819 ng/mL of urine (Cot, smokers). A total of 129 students agreed to take part in the study; 27 of them smoked cigarettes, and 65 admitted to being passive smokers. According to the questionnaire data filled out by the study participants, the remaining individuals (n = 37) did not smoke cigarettes and were not exposed to tobacco smoke either. The results of Cot, 3′-OH-Cot, and 2′S-Nic-Ox determinations in urine samples are shown in Table 4. The data correspond to the average of three LC-MS/MS analyses conducted for extracts obtained in duplicate for each urine sample.

**Figure 3:**
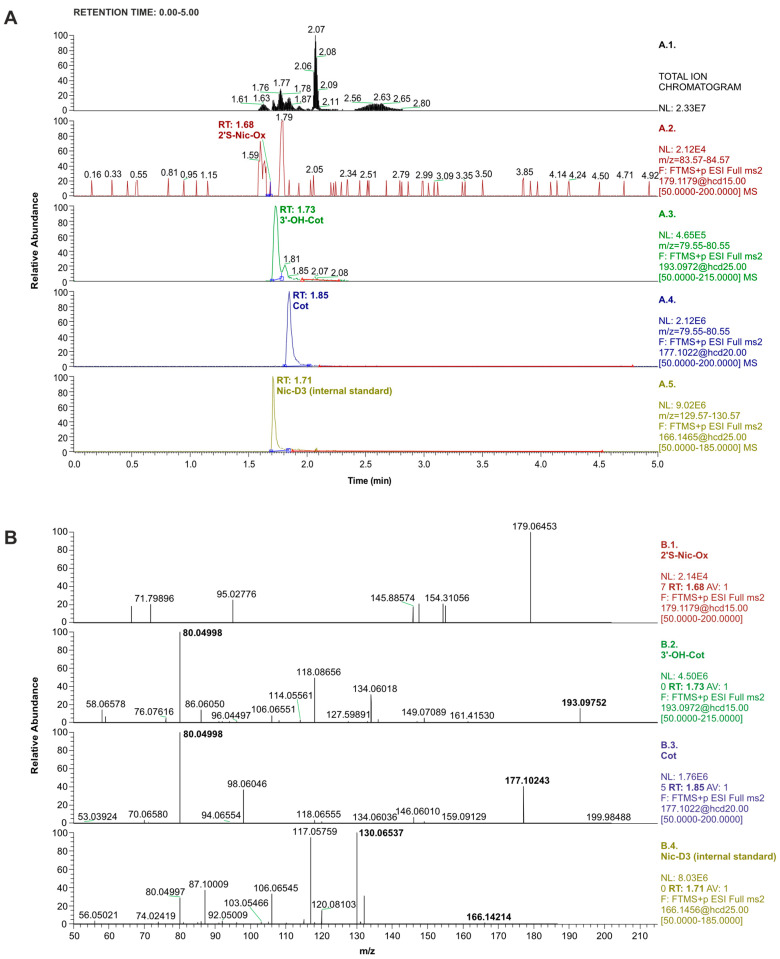
(**A**): LC-orbitrap-MS/MS chromatograms recorded for the smoker’s urine extract in total ion mode (top window) and PRM mode (consecutive windows from the top corresponding to 2′S-Nic-Ox, 3′-OH-Cot, Cot, and Nic-D3). The concentration of Nic-D3 was 0.3 µg/mL (injection volume: 1 µL). (**B**): Mass spectra recorded in PRM mode for analytes determined in the smoker’s urine extract. Consecutive windows from the top correspond to 2′S-Nic-Ox (not detected in this sample), 3′-OH-Cot, Cot, and Nic-D3 (internal standard). The corresponding collision energies are shown in Table 2.

The concentrations of Cot and 3′-OH-Cot determined in the urine of cigarette smokers were 70- and 58-fold higher, respectively, compared to passive smokers. On the other hand, higher concentrations of Cot and 3′-OH-Cot were recorded in the urine of passively exposed individuals than in non-smokers, confirming that passive exposure to tobacco smoke is not harmless to the human body. However, no differences were observed in the averaged concentration of 2′S-Nic-Ox in the urine samples of the different groups of subjects. In numerous samples, even from cigarette smokers, this nicotine metabolite was not detected. Figure 3A shows an example of total and PRM-mass chromatograms recorded during the analysis of a smoker’s urine sample. Figure 3B shows the mass spectra of Cot and 3′-OH-Cot determined in this sample. 2′S-Nic-Ox was not detected in this sample because the mass spectrum does not contain the characteristic product ion (*m*/*z* = 80.04999) formed from the precursor ion.

The mean urinary cotinine concentration of passive smokers was 55 ng/mL (Table 4). According to Kim, a range of urinary cotinine values of 50–200 ng/mL may serve as a cutoff point for verification of active smokers [32]. A previous study conducted by our team to determine the main metabolites of nicotine by ELISA technique also showed that in the group of active smokers, the concentration of the main metabolites of nicotine was higher than 200 µg/mL of urine. In the group of passive smokers, the concentration of main nicotine metabolites ranged from 20 to 200 µg/mL of urine, and in the group of non-exposed smokers, the concentration of major nicotine metabolites was lower than 20 µg/mL of urine [17]. 

High concentrations of nicotine metabolites were expected in students who smoked cigarettes. The total content of Cot and 3′-OH-Cot measured in urine was almost 6700 ng/mL in this group of subjects. These data correspond to values determined in urine samples by other authors [4,8,16,22,33,34,35]. In passive smokers, the mean summed concentration of these two nicotine metabolites determined at almost 106 ng/mL was three times higher than in non-smokers. It is worth noting that Cot was not determined in the urine of some passive smokers, while 3′-OH-Cot was present in these samples. A large-scale Korean National Health and Nutrition Examination Survey confirmed that cotinine may be absent from urine samples, although other nicotine metabolites were detected in them [33]. Previous work by our research group also confirmed that Cot may not be present in the urine of people exposed to tobacco smoke [20]. 

The opportunity to demonstrate to young people, especially medical students, that smoking cigarettes in the presence of others can lead to the accumulation of harmful compounds in their bodies is perhaps the only way to prevent civilization diseases resulting from smoking in the future. Of even greater concern is the carefree approach to the use of e-cigarettes by increasingly younger people [27,35,36,37]. The developed easy procedure of nicotine metabolites isolation from biological samples combined with a sensitive, rapid, and reliable determination method of LC-Orbitrap-MS/MS makes it possible to perform such tests during laboratory classes with students.

Few examples are available in the literature of the use of this analytical system for the determination of nicotine metabolites in biological samples, where their concentrations may be low.

The LC-orbitrap-MS/MS method was validated and used (in the SIM mode) for the determination of nicotine metabolites, Cot and 2′S-Nic-Ox, in urine [7] as well as in human plasma, semen, and sperm [31]. The chromatographic separation parameters were analogous to ours. Due to the more complex matrix of plasma and sperm samples, the authors used a methanol solution with trichloroacetic acid for extraction combined with protein precipitation. 

Kawasaki et al. used the LC-Orbitrap-MS/MS to determine Cot in the urine of quitting smokers [38] and Cot and 3′-OH-Cot in urine as biomarkers of exposure to secondhand smoke and heated tobacco products [39]. Due to the simultaneous determination of 4-(methyl-nitrosamino)-1-(3-pyridyl)-1-butanol, urine samples were subjected to β-glucuronidase treatment followed by liquid-solid extraction using columns containing diatomaceous earth. The resulting extracts, after evaporation and dissolution in acetonitrile containing 10 mM ammonium acetate, were analyzed by LC-orbitrap-MS/MS technique in PRM mode, to quantify the most intense product ion of each compound [38,39]. 

Taking advantage of the high selectivity sensitivity of LC-Orbitrap-MS/MS, a validated bioanalytical method was also developed for the simultaneous determination of nicotine and Cot in human blood based on the dry blood spot (DBS) technique after a single extraction step [40]. The calibration range established in this validated method for Cot was linear in the range of 0.010–0.500 µg/mL, which is similar to our work.

## 3. Materials and Methods

### 3.1. Reagents and Standards

Pierce™ LTQ Velos ESI Positive Ion Calibration Solution (Thermo Scientific, Rocford, IL, USA) was used to tune and calibrate the mass spectrometer. 

Organic solvents: acetonitrile and methanol, both hypergrade for LC-MS, as well as water for LC-MS were purchased from Merck (Darmstadt, Germany). Formic acid for LC-MS LichropurTM used as a mobile phase component was obtained from Sigma-Aldrich (St. Louis, MO, USA).

Four analytical standards were used in the study: DL-nicotine-(methyl-D3) (purity 99%) and (-)-cotinine (purity 98%) which were purchased from Sigma-Aldrich (St. Louis, MO, USA), *trans*-3′-hydroxycotinine (solution, 1 mg/mL methanol) was from Supelco (Round Rock, TX, USA) and (1′S,2′S)-nicotine 1′-oxide bought from Toronto Research Chemicals (Toronto, ON, Canada). Structures and names abbreviation of these compounds are presented in Table 1.

Standard stock solutions of Cot, 3′-OH-Cot, and 2′S-Nic-Ox (each 1.0 mg/mL) were used to prepare a standard mixture of concentration 1 µg/mL in acetonitrile. By diluting this standard mixture, solutions at concentrations of 0.75, 0.50, 0.25, 0.125, 0.060, 0.030, 0.015, and 0.010 µg/mL were prepared and used to create calibration curves and establish detection limits. DL-nicotine-(methyl-D3) in acetonitrile was introduced into each solution as an internal standard, in an amount that referred to a final concentration of 0.30 µg/mL.

### 3.2. Urine Sample Collection and Extraction

Urine samples were collected from volunteers who were smokers, passively exposed to tobacco smoke, and non-smokers. The samples were centrifuged and stored in a freezer at −20 °C until analysis. The study was conducted among 129 first- and second-year medical and emergency medicine students. The mean age of the study group was 21.1 ± 1.3 years, 21.2 ± 1.4 years for women and 21.1 ± 1.2 years for men. Along with urine samples, survey data were collected using a self-prepared questionnaire designed to assess exposure to tobacco smoke. To conduct such research Approval of the bioethics committee has been obtained (No BNW/NWN/0052/KB1/81/23 from 12.09.2023). 

To extract nicotine metabolites, 0.5 mL of urine sample was transferred to an Eppendorf tube and 1 mL of acetonitrile containing 0.45 µg of Nic-D3 (as internal standard) was added. The samples were vortexed for 1 min and then centrifuged for 10 min at 4000 rpm using laboratory centrifuge MPW-260 R (MPW med.Instruments, Warsaw, Poland). Extraction of each sample was carried out in two replicates. Each supernatant was transferred into a 2-mL autosampler vial and analyzed by LC-Orbitrap-MS/MS technique.

### 3.3. Determination of Nicotine Metabolites by LC-Orbitrap-MS/MS

A Vanquish Thermo Scientific liquid chromatograph assembled with a double split sampler, column thermostat compartment, and double pump (all from Thermo Scientific, Germering, Germany) was used. For the separation of urine extracts components an analytical column Accucore-C18 (150 × 3 mm I.D.; particle size 2.6 μm) from Thermoscientific (Lithuania) was applied. The separations were performed under isocratic conditions by using a mixture of acetonitrile, water, and formic acid (10:90:0.1, *v*/*v*/*v*) as a mobile phase at a constant flow rate of 0.3 mL/min. The temperature of the automatic sampler tray was set to 10 °C, and the temperature of the column thermostat was set to 30 °C. The injection volume of standard solutions and extracts was 1 µL.

The mass spectrometer system connected with the liquid chromatograph consisted of an Orbitrap mass spectrometer (Q Exactive Focus, Thermo Fisher Scientific, Bremen, Germany), equipped with a heated electrospray ionization (HESI) source. The system was controlled by Xcalibur LC Devices 3.2 Robust software.

The mass spectrometer operated in full MS-SIM and secondary mass (MS/MS) monitoring in positive ion mode for the analytes to be determined. The orbitrap was tuned and calibrated using the ESI positive ion calibration solution once a week.

The nitrogen gas for the ion source was produced by the generator Nigen LCMS 40-1 (Claind Brezza, Tremezzina, Italy). The sweep, auxiliary, and sheath gases flow were set at 5, 20, and 45 units, respectively. The ion spray voltage was set at 3.5 kV and the temperature of the ion transfer capillary was set at 310 °C. The MS resolution option was 17.5 at 2 Hz with a mass window of 0.4 Da for each analyte.

The determination of Cot, 3′-OH-Cot, and 2′S-Nic-Ox was performed in parallel reaction monitoring (PRM) mode. The optimal collision energies used to obtain product ions from precursor molecular ions [M+H]^+^ corresponding to the maximum intensity for the analytes are shown in Table 2. These values were selected based on a series of analyses performed at different collision energies for standard solutions of a concentration of 0.25 µg/mL.

### 3.4. Calibration Graphs, Limits of Detection and Limits of Quantification, Repeatability and Reproducibility, and Recovery of Extraction 

Quantitative analysis of Cot, 3′-OH-Cot, and 2’S-Nic-Ox was performed on the basis of linear calibration graphs recorded in the range of 0.03 to 1.0 µg/mL, for 1 µL of standard mixture injected into the column (Table 3). Each calibration solution contained Nic-D3 as the internal standard at the concentration of 0.3 µg/mL. The graphs were plotted on the ratio of the peak area recorded for the appropriate analyte to the peak area corresponding to IS, relative to the analyte concentration in the standard mixture. The regression coefficients R for the curves were above 0.996. 

Limits of detection (LODs) were determined using the stepwise dilution method of standard solutions, taking them as a signal-to-noise (S/N) ratio of 3. A value of 3 times the limit of detection (LOD) was taken as the limit of quantification (LOQ) for an injection volume of 1 µL [41]. They were 0.01 and 0.03 µg/mL, respectively, for all compounds selected for the study, i.e. for Cot, 3′-OH-Cot, and 2′S-Nic-Ox.

The procedure to determine recovery levels was conducted using urine from a non-smoker. The urine was enriched with standards of nicotine metabolites at three concentration levels (i.e., 0.05, 0.5, and 1.0, with six replicates at each level), as well as the internal standard. The samples prepared this way were subjected to extraction, and then the analytes were determined by LC-orbitrap-MS/MS in PRM mode. Recovery values are shown in Table 3. They ranged from 72.2% to 101.4%. The formula [(C1 − C2)/C]∙100% was used to calculate the recovery, where C1 is the labeled analyte concentration in the urine sample with standards added (µg/mL), C2 is the determined concentration in the urine sample (µg/mL) not enriched with standards, and C is the amount of µg standard added to 1 mL of urine sample. The repeatability (intra-day precision) of the method was assessed by the relative standard deviations (RSD) of the nicotine metabolite determinations obtained on the same day (n = 5) for samples enriched with standards. These ranged from 1.0 % to 3.1%. The inter-day precision (reproducibility) over 5 days ranged from 3.0% (3′-OH-Cot, 0.05 µg/mL) to 10.1% (2′S-Nic-Ox, 1 µg/mL).

## 4. Conclusions

The procedure for precipitation of proteins with acetonitrile is a very simple and quick technique for preparing biological samples to remove components that can interfere with LC-MS detection. The use of an internal standard allows better control of this process. Combined with LC-orbitrap-MS/MS analysis, in parallel reaction monitoring (PRM) mode, it was possible to determine selected nicotine metabolites (cotinine, *trans*-3′-hydroxycotinine and (1′S,2′S)-nicotine 1′-oxide) in urine samples of smokers and passive smokers using the fast cleanup procedure with acetonitrile. Extraction recoveries of these analytes from urine ranged from 72% (2′S-Nic-Ox) to 101% (Cot and 3′-OH-Cot).

The total determined Cot and 3′-OH-Cot concentration in the urine samples of cigarette smokers was 6786 ng/mL, whereas in samples of participants passively exposed to tobacco smoke, the concentration was only 106 ng/mL, but still 3 times higher than in non-smokers not exposed to cigarettes.

Although metabolite concentrations in passively exposed individuals were much lower than in smokers, it still raises the question of concern of neglecting, by young adults and additionally medicine students, the potential sources of exposure to harmful chemicals and tolerating smoking in the company of non-smokers. It also shows how important it is to implement healthy habits at the early stage of adult life, especially in the population of future physicians who are supposed to promote pro-health attitudes.

While many countries ban the use of tobacco products in public places (restaurants, public transport, public beaches, outdoor swimming pools), newly introduced tobacco products such as e-cigarettes may pose other health risks. In recent years, the use of e-cigarettes has become fashionable among young people because of their perceived safety and the widespread belief that they can help smokers quit traditional cigarettes and minimize withdrawal symptoms. It is also believed that e-cigarettes do not contain many harmful substances [8,26]. Our future research goal is to implement the method described in this paper to analyze urine samples of young adults and medical students who use e-cigarettes, and individuals passively exposed to e-cigarette smoke.

## Figures and Tables

**Table 1:** Structures, names, and molecular formulas of the compounds used in this study.

Name (Abbreviation)	Structure	Molecular Formula	Molecular Mass
(-)-Cotinine (Cot)	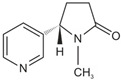	C_10_H_12_N_2_O	176.09496
*trans*-3′-Hydroxycotinine (3’-OH-Cot)	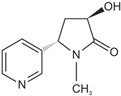	C_10_H_12_N_2_O_2_	192.08988
(1′S,2′S)-nicotine 1′-oxide (2’S-Nic-Ox)	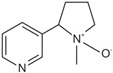	C_10_H_14_N_2_O	178.110161
DL-nicotine-(methyl-D3) (Nic-D3)	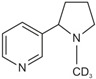	C_10_D_3_H_11_N_2_	165.134528

**Table 2:** Parameters for the analysis of nicotine metabolites and internal standard by LC-Orbitrap-MS/MS in parallel reaction monitoring (PRM) mode.

Compound	Retention Time [min]	Precursor Ion [M+H]^+^ → Product Ion (*m*/*z*)	Collision Energy (eV)
Cot	1.85	177.10240 → 80.04999	20
3′-OH-Cot	1.72	193.09727 → 80.04999	25
2′S-Nic-Ox	1.68	179.11810 → 84.08128	15
Nic-D3	1.72	166.14189 → 130.06540	25

**Table 3:** Data from the determination of nicotine metabolites by LC-Orbitrap-MS/MS in PRM mode.

Nicotine Metabolite	Calibration Curve Concentration Range (µg/mL)	Regression Coefficients *r*	Recovery (%) and RSD ^1^ (%) for Spiking Level (µg/mL of Urine); n = 6		
			0.05	0.5	1.0
Cot	0.03–1.00	0.9980	101.4 (1.0)	82.2 (1.0)	81.1 (1.1)
3′-OH-Cot	0.03–1.00	0.9965	100.9 (2.0)	85.6 (1.0)	83.6 (1.0)
2′S-Nic-Ox	0.03–1.00	0.9979	82.8 (1.6)	75.0 (3.1)	72.2 (2.5)

^1^ RSD—relative standard deviation.

**Table 4:** Nicotine metabolites concentration (ng/mL of urine)

Nicotine Metabolite	Status of Smoking		
	Smokers (Min–Max) ^1^	Passive Smokers (Min–Max)	Non-Smokers (Min–Max)
Cot	3819.0 (639.0–12,540.0)	54.6 (n.d.–94.1)	n.d. ^2^
3′-OH-Cot	2967.0 (261.0–7080.0)	51.1 (36.0–137.5)	35.4 (0–36.0)
2′S-Nic-Ox	55.4 (n.d.–90.0)	53.4 (n.d.–77.8)	53.7 (0–54.0)
Cot + 3′-OH-Cot	6786.0	105.7	35.4

^1^ Concentration range (from minimum to maximum concentration determined); ^2^ n.d. -not determined (below the limit of quantification (LOQ).

## Data Availability

The raw data supporting the conclusions of this article will be made available by the authors on request.

## Supplementary Materials

The following supporting information can be downloaded at: https://www.mdpi.com/article/10.3390/molecules29153643/s1, Figure S1A. Total and selected ion chromatograms (SIM) of nicotine metabolites in standard mixture and of internal standard; Figure S1B. Total and selected ion chromatograms (SIM) of nicotine metabolites in urine from a cigarette smoker; Figure S1C. Total and selected ion chromatograms (SIM) of nicotine metabolites in urine from a passive smoker; Figure S1D. Total and selected ion chromatograms (SIM) of nicotine metabolites in urine from a non-smoker.
