# Supplementary material for: Determination of Cotinine, 3′-Hydroxycotinine and Nicotine 1′-Oxide in Urine of Passive and Active Young Smokers by LC-Orbitrap-MS/MS Technique
Source: Molecules. 2024 Aug 1;29(15):3643. doi: 10.3390/molecules29153643 (PMC11313786; doi:10.3390/molecules29153643)
Supplement: Supplementary file 1 [file molecules-29-03643-s001.zip › molecules-3133040-supplementary.pdf]

Supplemental data S1

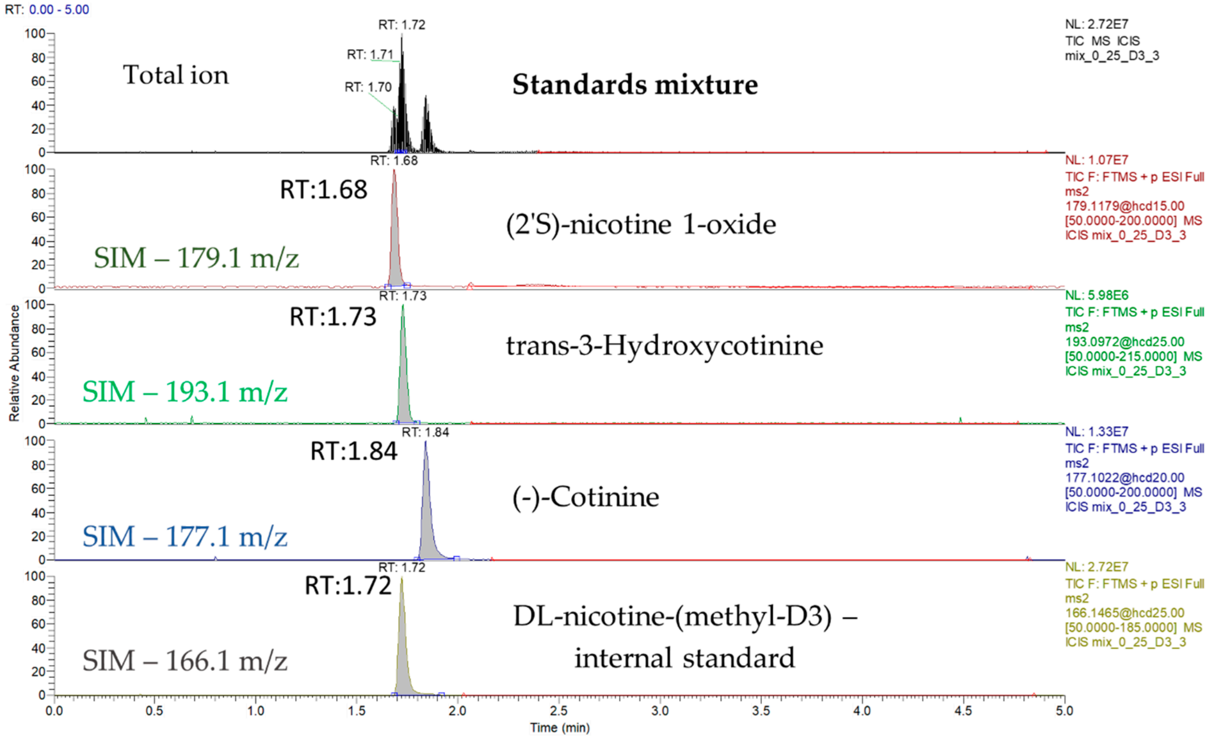

Figure S1 (A). Total and selected ion chromatograms (SIM) of nicotine metabolites in standard mixture and of internal standard

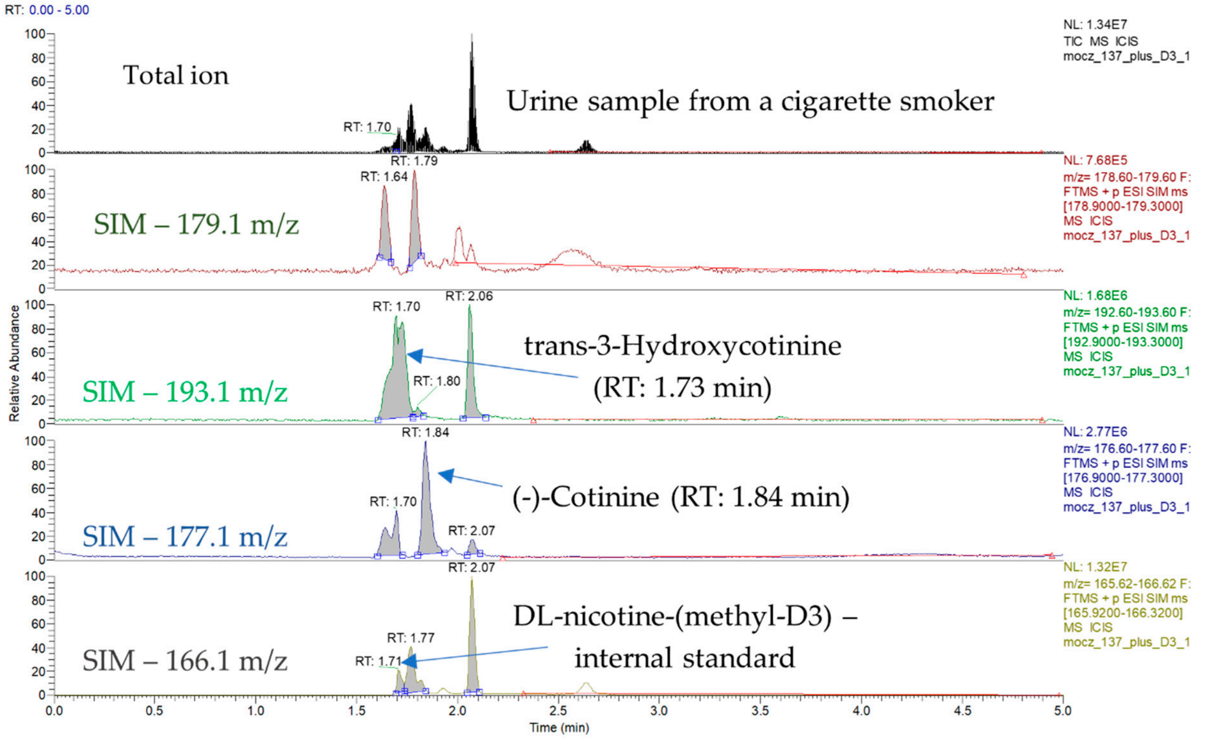

Figure S1 (B). Total and selected ion chromatograms (SIM) of nicotine metabolites in urine from a cigarette smoker

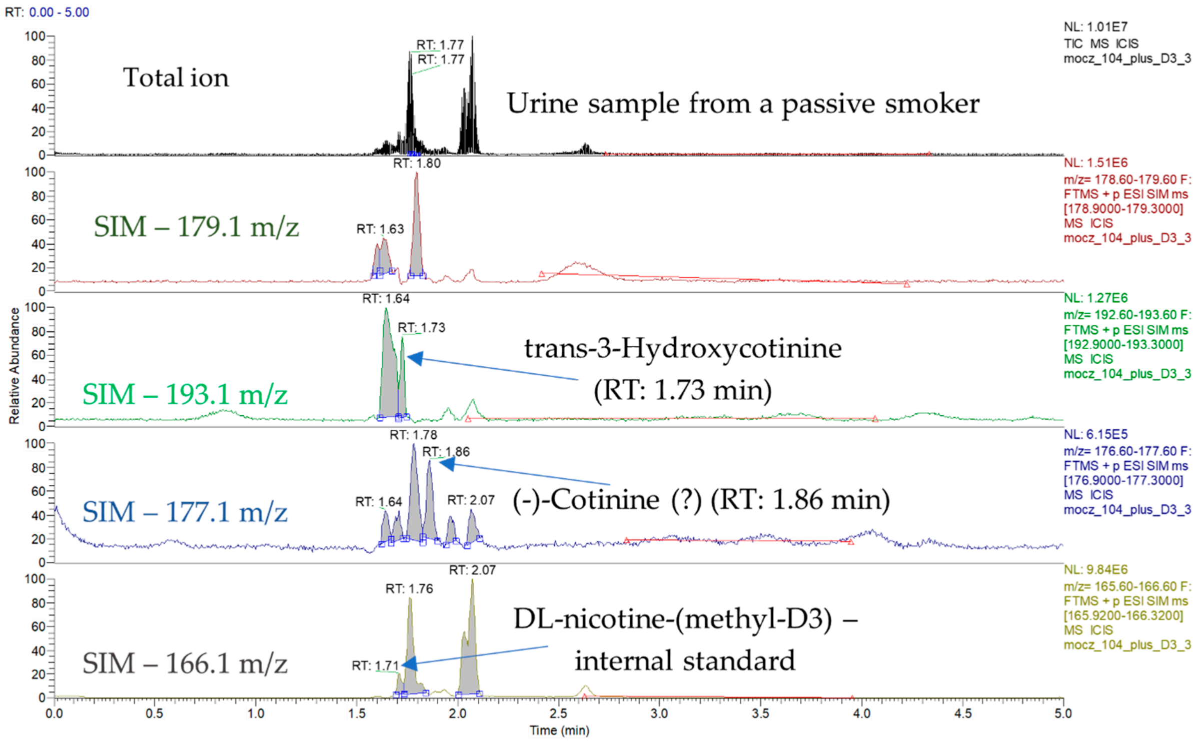

Figure S1 (C). Total and selected ion chromatograms (SIM) of nicotine metabolites in urine from a passive smoker

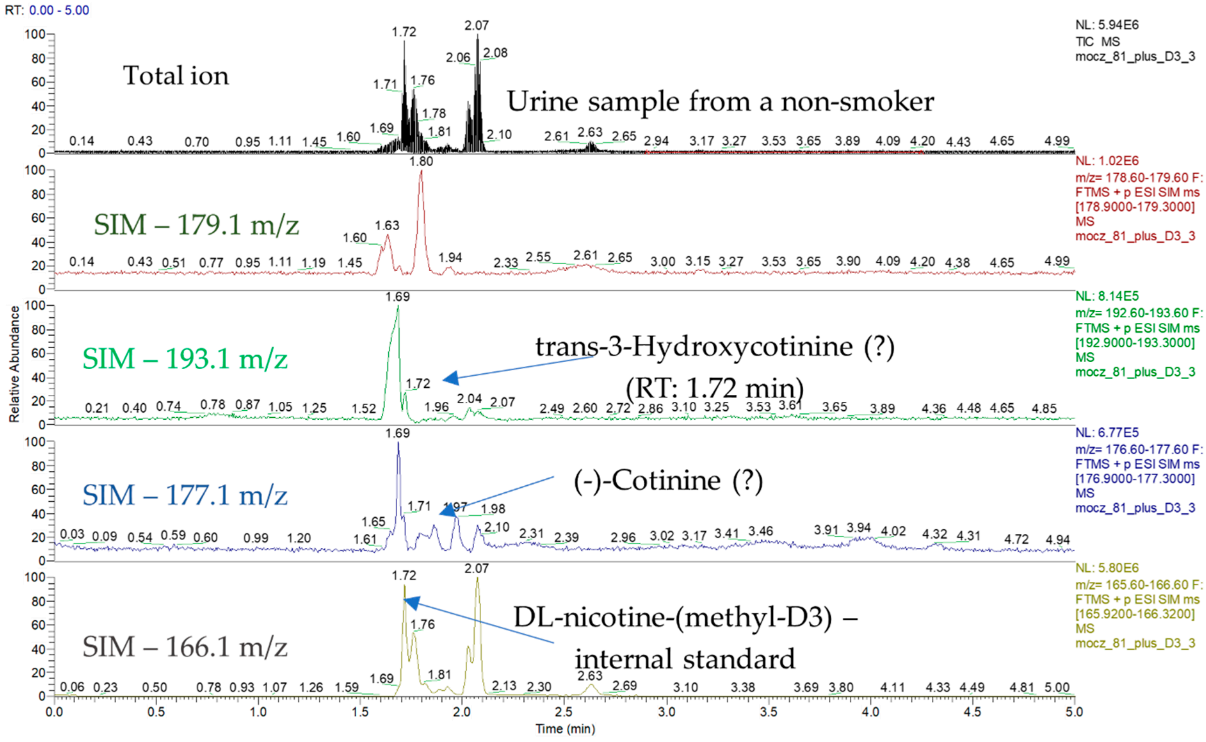

Figure S1 (D). Total and selected ion chromatograms (SIM) of nicotine metabolites in urine from a non-smoker
